# Neural Correlates of Auditory Hypersensitivity in Fragile X Syndrome

**DOI:** 10.3389/fpsyt.2021.720752

**Published:** 2021-10-07

**Authors:** Khaleel A. Razak, Devin K. Binder, Iryna M. Ethell

**Affiliations:** ^1^Department of Psychology, University of California, Riverside, Riverside, CA, United States; ^2^Graduate Neuroscience Program, University of California, Riverside, Riverside, CA, United States; ^3^Division of Biomedical Sciences and Graduate Biomedical Sciences Program, School of Medicine, University of California, Riverside, Riverside, CA, United States

**Keywords:** Fragile X Syndrome, autism spectrum disorders, sensory processing disorders, auditory processing, sensory hypersensitivity, matrix metalloproteinase, GABA

## Abstract

The mechanisms underlying the common association between autism spectrum disorders (ASD) and sensory processing disorders (SPD) are unclear, and treatment options to reduce atypical sensory processing are limited. Fragile X Syndrome (FXS) is a leading genetic cause of intellectual disability and ASD behaviors. As in most children with ASD, atypical sensory processing is a common symptom in FXS, frequently manifesting as sensory hypersensitivity. Auditory hypersensitivity is a highly debilitating condition in FXS that may lead to language delays, social anxiety and ritualized repetitive behaviors. Animal models of FXS, including *Fmr1* knock out (KO) mouse, also show auditory hypersensitivity, providing a translation relevant platform to study underlying pathophysiological mechanisms. The focus of this review is to summarize recent studies in the *Fmr1* KO mouse that identified neural correlates of auditory hypersensitivity. We review results of electroencephalography (EEG) recordings in the *Fmr1* KO mice and highlight EEG phenotypes that are remarkably similar to EEG findings in humans with FXS. The EEG phenotypes associated with the loss of FMRP include enhanced resting EEG gamma band power, reduced cross frequency coupling, reduced sound-evoked synchrony of neural responses at gamma band frequencies, increased event-related potential amplitudes, reduced habituation of neural responses and increased non-phase locked power. In addition, we highlight the postnatal period when the EEG phenotypes develop and show a strong association of the phenotypes with enhanced matrix-metalloproteinase-9 (MMP-9) activity, abnormal development of parvalbumin (PV)-expressing inhibitory interneurons and reduced formation of specialized extracellular matrix structures called perineuronal nets (PNNs). Finally, we discuss how dysfunctions of inhibitory PV interneurons may contribute to cortical hyperexcitability and EEG abnormalities observed in FXS. Taken together, the studies reviewed here indicate that EEG recordings can be utilized in both pre-clinical studies and clinical trials, while at the same time, used to identify cellular and circuit mechanisms of dysfunction in FXS. New therapeutic approaches that reduce MMP-9 activity and restore functions of PV interneurons may succeed in reducing FXS sensory symptoms. Future studies should examine long-lasting benefits of developmental vs. adult interventions on sensory phenotypes.

## Introduction

There is a strong association between autism spectrum disorders (ASD) and sensory processing disorders (SPD). Indeed, the latest diagnostic criteria for ASD includes atypical sensory function as a core deficit. Research findings in both humans with ASD and animal models of ASD suggest that abnormal sensory processing in early development may lead to a broader array of symptoms including abnormal anxiety, social, and hyperactive behaviors (1–5). Despite the association between ASD behaviors and SPD, little is known about underlying cellular and circuit mechanisms that links autism to sensory issues. This review focuses on recent studies of the auditory system in Fragile X Syndrome (FXS), the most common genetic cause of ASD-associated behaviors and makes the case that studying basic sensory processing has multiple advantages in terms of identifying translation-relevant neural correlates, while at the same time gaining insight into the circuit mechanisms that lead to symptoms.

## Fragile X Syndrome

Fragile X syndrome is a genetic disorder that affects ~1 in 4,000 males and 1 in 8,000 females (6, 7). FXS results from the loss of Fragile X Mental Retardation protein (FMRP), an mRNA binding protein that targets key synaptic pathways. FMRP is reduced or absent in humans with FXS due to an expansion and hyper-methylation of CGG trinucleotide repeats in the promoter region of the *FMR1* gene (8). Individuals with FXS experience a wide array of symptoms including intellectual impairment, language delays, seizures, repetitive behaviors, social anxiety, and hyperactivity. Consistently, abnormal sensory sensitivity (typically hypersensitivity) is seen in humans with FXS. Approximately 15–33% of individuals with FXS meet the diagnostic criteria for autism, with ~5% of autism cases attributed to FXS (9–12). Many symptoms of FXS and ASD are similar, suggesting that studies of neural mechanisms in FXS may be broadly informative.

## Why Study the Auditory System in FXS?

Both humans with FXS, and a commonly used animal model of the condition, the *Fmr1* knockout (KO) mouse, show auditory hypersensitivity. Neural circuits involved in auditory processing, particularly those in the early stages of processing, are likely to be more conserved across humans and rodents than circuits involved in social and cognitive symptoms. There are many similarities between humans and rodents in the basic organization of the auditory system from subcortical areas to the primary auditory cortex. There is also a rich history of studying auditory system development. Given that FXS is a neurodevelopmental disorder, existing knowledge on normal auditory circuit development provides a strong basis to study circuits that underlie hypersensitivity. The auditory system and auditory-related symptomatology offer a translation-relevant platform to identify clinically-relevant phenotypes and study circuit mechanisms of deficits in FXS. Indeed, as reviewed below, studies of auditory cortical processing in humans with FXS and mouse models have found remarkable similarities across species.

## EEG Phenotypes Related to Sensory Processing in Humans With FXS

Many of the early studies of auditory hypersensitivity in humans with FXS focused on auditory event-related potential (ERP) recordings. ERP studies consistently showed enhanced amplitude of various components (e.g., N1, P2). Enhanced synchrony of population responses to individual tones is likely responsible for enlarged N1 component of ERPs observed in humans with FXS (13–20), which may be generated by specific cell types in the auditory and frontal cortex (21, 22). A study using MEG also revealed enlargement of the N100m [the MEG equivalent of the N1 in EEG (14)]. In addition, the habituation of the N1 component to repeated tones is reduced in humans with FXS (17, 23); and the P2 amplitude of the ERP is enhanced in FXS (18). The similarity in observed MEG and EEG phenotypes adds further validity to the findings. The increase in N1 and P2 amplitude may be related to neuroanatomical abnormalities in the superior temporal gyrus (STG) where the auditory cortex is located (24), and to white matter enlargement in the temporal lobe (25). The enhanced N1 amplitude is also consistent with functional imaging studies that show that the STG displays higher levels of activation in individuals with FXS (26). Behavioral auditory hypersensitivity may therefore result from altered cortical responses to sounds (cortical hyperexcitability) in humans with FXS. Both enhanced population responses to sounds and reduced habituation of cortical neurons to repeating sounds may lead to auditory hyperexcitability (27).

### Human EEG Spectral Component Analysis and Relationship to Clinical Measures

More recent EEG studies in humans have examined spectral components of baseline and sound-evoked responses to identify deficits in neural oscillations that are associated with sensory and cognitive symptoms in FXS. Wang et al. (28) found that FXS patients (*n* = 21, mean age = 26.4, range 10–55 yrs) exhibited greater gamma frequency band power (30–80 Hz) in the resting state EEG compared to age matched controls (*n* = 21). There was a reduction in alpha-gamma amplitude coupling across electrodes in FXS that suggests reduced top-down cortico-cortical control in FXS (29). The gamma power abnormality was correlated with social and sensory processing difficulties as measured with Social Communication Questionnaire and Adolescent/Adult Sensory Profile scores. These data are consistent with the reduced alpha-increased gamma power trends observed across ASDs (30). Ethridge et al. (31) replicated the gamma band finding in humans with FXS (*n* = 17, mean age = 26.2, range = 11–55) and showed that abnormalities in gamma power were related to more severe behavioral and psychiatric features and reductions in neurocognitive functions. In addition, test-retest data shows reliability of measures in a third group of humans with FXS (*n* = 38, mean age = 25.5, range = 10–53) (18). Taken together, the resting EEG gamma power and ERP amplitude phenotypes have been replicated multiple times, with indications of scalability and retest reliability, which is critical for biomarker development. Importantly, these data demonstrate a close relationship between EEG measures and clinical manifestations.

Although elevated gamma power is found consistently, additional studies are needed to address its relevance. For example, Wilkinson and Nelson (32) found elevated aperiodic power in the beta-gamma range (25–50 Hz) in a younger cohort of boys with FXS age 2.5–7 (mean ~4 yrs). However, they found no association between gamma power and sensory hypersensitivity or adaptive behaviors. Rather, they found an association between elevated gamma power and improved language ability in boys with FXS, suggesting that the gamma elevation may reflect compensatory mechanisms in FXS (33). Given the links between gamma oscillations and sensory-cognitive functions, and the emerging evidence that aperiodic gamma power may reflect cortical activation and excitatory/inhibitory balance in the cortex (34), a comprehensive quantification of oscillatory and aperiodic gamma power in the resting EEG needs to be obtained and correlated with clinical scores across development to properly identify biomarkers for clinical use. Abnormal periodic and aperiodic gamma power may serve as specific biomarkers for stratification of patients and outcome measures for clinical trials.

The gamma power related to local network excitation may reduce the ability of the neural population to synchronize periodic gamma band activity. Indeed, Ethridge et al. (31) found specific deficits in the gamma synchronization by testing the ability of the neural generators of the EEG signals to phase lock to dynamic auditory stimuli called “chirp.” The chirp stimulus is a tone whose amplitude is modulated by a sinusoid of linearly increased or decreased frequency in the 1–100 Hz range. The ability of the auditory system to phase lock consistently across trials to the different frequencies (1–100 Hz range) in the chirp is quantified as the inter-trial phase coherence (ITPC). Humans with FXS including ages in 12–57 range (mean ~26 yrs) show reduced ITPC in the 30–50 Hz gamma frequencies, but enhanced non-phase locked baseline broadband gamma power as the chirp trial was ongoing. These findings were replicated in another study of control and FXS human subjects (mean ~25 yrs, range 10–53) at a different clinical site and using different EEG equipment (18) compared to the Ethridge et al. (31) paper.

The abnormal responses of auditory cortex to sound that are present from early development may affect communications and language skills (35, 36). Indeed, humans with FXS show delays and abnormalities in expressive language skills [Reynell Developmental Language Scales—Roberts et al. (37)]. Individuals with FXS experience difficulty articulating words, poor co-articulation, substitutions, and omissions of words, reduction in the number of intelligible syllables produced, difficulty sequencing sounds, and echolalia (38–42). Similar language delays seen in autism may be associated with basic auditory processing abnormalities in early sensory cortical regions (43). Schmitt et al. (44) used a “talk/listen” paradigm and EEG recordings to address possible underpinnings of the expressive language deficits in FXS. In this task, EEGs were recorded when the subject either uttered a phoneme or passively listened to the same phoneme. In a healthy individual a suppression of ERP component amplitudes is normally observed when subjects say the phoneme compared to when they listen to it (so called N1 suppression) with a negative signal in the EEGs just before the speech sound is produced (pre-speech negativity). These changes are attributed to an efference copy from the motor generators to the speech perception regions of the brain. In contrast, FXS subjects showed reduced pre-speech negativity and elevated gamma power in frontal loci that were related to speech intelligibility when frontal and temporal EEG recordings were compared between controls and humans with FXS (44). There was also reduced frontotemporal coherence in the theta-alpha frequency bands just prior to speech production, but no difference in N1 suppression was observed during the speech production. These EEG data suggest that abnormal signaling between frontal and temporal cortical regions (45) may underlie the expressive speech deficits in FXS. Elevated gamma power in the pre-speech time window indicates the gamma phenotype described above in sensory regions is also seen more broadly, can be task-related, and may relate to broader cognitive deficits in FXS.

## EEG Phenotypes Related to Sensory Processing in Animal Models of FXS

Recent implementation of new EEG technology for pre-clinical studies in awake and freely moving mice demonstrated that similar EEG phenotypes are also observed in animal models of FXS, mainly *Fmr1* KO mice (Table 1) (51, 58). Lovelace et al. (55) compared EEG recordings between adult WT and *Fmr1* KO mice on FVB background and showed elevated baseline gamma power, reduced phase locking at gamma band frequencies with the chirp stimuli, enhanced non-phase gamma band power during the chirp trials and enhanced N1 ERP amplitude. Enhanced gamma power, enhanced ERP amplitude and reduced gamma synchronization to chirp are also seen in adult *Fmr1* KO mice on the C57BL6 background (47, 55). In addition, enhanced baseline gamma power and impaired sound-responses were observed in young P21–P28 *Fmr1* KO mice from both backgrounds (52, 59), suggesting early development of the abnormal EEG phenotypes. Interestingly, the *Fmr1* KO mice showed a larger increase in gamma band power during movement (46), suggesting the possibility that the motor modulation of auditory cortex may be abnormal in FXS. Although abnormal habituation to sound was not reported in awake and freely moving mice, earlier EEG studies in anesthetized adult *Fmr1* KO mice on the FVB strain showed reduced habituation of N1 with repeated stimulation (56). This phenotype has not been tested in younger mice or the C57 strain. While these EEG data were obtained with epidural screw electrodes, for more immediate translation relevance, recent studies using a 30-channel skull surface multielectrode array (MEA) recording technique showed essentially the same EEG phenotypes in the *Fmr1* KO mice (49). The increased number of recording sites, along with broader spatial coverage will now facilitate advanced EEG analysis, including cross-frequency and cross-region analysis in awake and freely moving mice to more closely relate to high-density human EEG studies.

**Table 1:** Species similarity in EEG phenotypes.

**EEG phenotype**	***Fmr1* KO mouse/rat**	**Humans with FXS**
Resting (baseline) EEG gamma band power	Increased/increased (46–54)	Increased (18, 28, 30, 31)
Non-phase-locked power in the gamma band	Increased (47, 48, 50, 52)	Increased (18, 30, 31)
ERP N1 amplitude	Increased (46, 49, 50, 55, 56)	Increased (13, 15, 16, 19)
ERP N1 habituation	Decreased (56)	Decreased (18, 23)
Phase locking to chirp stimuli in the gamma band (ITPC)	Decreased (46–49, 54)	Decreased (18, 31)
Phase locking in 40 Hz auditory steady state response (ITPC)	Decreased/decreased (48, 57)	*Non tested*
Cross-frequency coupling	Reduced alpha-gamma coupling (52)	Reduced alpha-gamma coupling (28)

*The table lists the major EEG findings in rodents and humans that could be used as EEG correlates of sensory hypersensitivity. The direction of change is remarkably similar across species. ITPC, Inter-Trial Phase Coherence*.

Similar EEG phenotypes were also observed in the *Fmr1* KO rat model of FXS, which displayed enhanced baseline gamma band power, reduced alpha power and behavioral hyperactivity (57). In addition, sound-evoked response, more specifically ITPC when tested with click trains to elicit an auditory steady state response, also showed a decrease in the gamma oscillations in the *Fmr1* KO rat. The findings were consistent with reduced ITPC auditory steady state response observed in the *Fmr1* KO mouse in response to a 40 Hz click train (47). Interestingly, studies in juvenile *Fmr1* KO rat visual cortex showed that the typical switch from higher to lower frequency dominance in cortical response was impaired when the animal went from an active to a resting state (53). The high-frequency power remained elevated in the *Fmr1* KO rat compared to the WT counterparts yet again suggesting abnormal modulation of sensory cortex responses by movement states. The species similarity (humans, mice, and rats) in the EEG phenotypes and the specific frequency bands affected is remarkable, and could prove critically useful in developing similar outcome measures between pre-clinical and clinical trials, while at the same time facilitate discovery of underlying cellular and circuit mechanisms, and new therapeutic interventions in the animal models. Future studies need to validate selected EEG phenotypes as biomarkers by performing studies on robustness, scalability, tolerance to settings and equipment and sensitivity to drug treatments.

## Systems, Circuit, and Cellular Mechanisms of Auditory Hypersensitivity in FXS

Considering clinical relevance of the sensory hypersensitivity, several recent studies are focused on deciphering cellular and circuit mechanisms underlying it, utilizing both *in vivo* and *in vitro* approaches. Rotschafer and Razak (60) showed that individual neurons in the auditory cortex of *Fmr1* KO mice responded with more action potentials to tones than in WT mice, using *in vivo* single unit recordings. Although the onset responses were similar across the genotypes, the responses were prolonged and continued well after sound offset in *Fmr1* KO neurons, but not in WT neurons. This indicates an increased duration of responses in the *Fmr1* KO mouse cortex, and may be related to the observed increase in baseline corrected single trial power (18, 46) and increase in resting gamma power in EEG responses (46, 50). Rotschafer and Razak (60) also showed that the frequency tuning receptive field of cortical neurons was broader in the *Fmr1* KO mice. This indicates that for the same tone, more neurons will be synchronously activated in the auditory cortex of *Fmr1* KO mice compared to WT mice and may underlie enhanced N1 amplitudes of ERPs, and the larger STG activation in humans with FXS (26). These increases in neural responses may arise from abnormal activation of inhibitory neurons (61, 62). In these studies, an examination of excitatory (E) and inhibitory (I) inputs to neurons in the somatosensory cortex provides important clues in terms of underlying circuit mechanisms of cortical neuron hyper-responsiveness. The strength of cortical E → E and I → E synaptic connections is shown to be relatively normal in the developing somatosensory cortex of *Fmr1* KO mice. However, cortical E → I synaptic communication is reduced leading to reduced activation of inhibitory neurons, that may lead to increased excitation in the network. Local hyperconnectivity between pyramidal neurons due to deficient pruning may also lead to increased synchrony and responses in the network (63).

### Development of Electrophysiological Abnormalities in *Fmr1* KO Mice

To investigate developmental trajectory of the abnormal phenotypes, Wen et al. (59) compared neuronal responses to sound between *Fmr1* KO and WT mice and identified the postnatal (P14–P21) window during which cortical responses began to diverge in the auditory cortex of *Fmr1* KO mice. Single unit recordings showed that responses were similar in cortical neurons of WT and *Fmr1* KO mice at P14. However, the responses were larger in the *Fmr1* KO cortex at P21. This indicates that just after hearing onset (~P10) in mice, the abnormal development of circuits induced by auditory experience may underlie cortical hypersensitivity in the *Fmr1* KO mice. The *Fmr1* KO rat visual cortex, as well, shows a divergence of responses around the period of eye-opening (53). The P14–21 developmental window coincides with the age during which the excitatory and inhibitory connections mature in the mouse auditory cortex acquiring adult-like characteristics (64, 65). Perturbation of auditory experience during this window using tone exposure leads to tonotopic plasticity in the WT mouse, but such critical period plasticity is disrupted in *Fmr1* KO mice (66), possibly due to impaired stability of long-term potentiation (67).

### Disentangling Cortical vs. Subcortical Contributions to Auditory Hypersensitivity

Besides auditory cortex, FMRP expression is detected across the entire auditory neuraxis, with the possible exception of the cochlea (68–70). While the preponderance of studies in both humans and animal models have focused on the cortex, both subcortical site abnormalities and/or local cortical processing abnormalities may contribute to the phenotypes recorded in the cortex (70–73). Indeed, both the brainstem and midbrain auditory nuclei show abnormal synaptic markers and electrophysiological responses. The inferior colliculus shows broader frequency tuning curves, and enhanced responses to tones and amplitude modulated sounds (73). As in the cortex, these abnormalities develop between P14 and P21, a time window during which intracollicular intrinsic inhibition matures to adult-like levels (74). More neurons exhibit cFos immunoreactivity in response to sounds in the inferior colliculus, indicative of enhanced cell activation, suggesting that population synchrony may be elevated in this region. The hyperexcitability of the inferior colliculus during early development is consistent with the suggestion that this midbrain region is involved in the generation of audiogenic seizures, a commonly studied phenotype in *Fmr1* KO mice (75). Supporting the role of midbrain in increased susceptibility to the audiogenic seizures, re-expression of FMRP in the glutamatergic neurons of inferior colliculus, in the Fmr1 KO mouse, prevents audiogenic seizures. Conversely, the deletion of Fmr1 in glutamatergic neurons of the inferior colliculus triggers audiogenic seizures. These data suggest that subcortical auditory sites show hyperexcitability, at least during early development.

While the brainstem and midbrain studies suggest that cortical hyperexcitability may reflect subcortical abnormalities, *in vitro* slice studies also indicate that local cortical processing may be abnormal. Goswami et al. (76) found that layer 2/3 circuits were hyperexcitable and showed increased gamma power in layers 2/3 and 5 in auditory cortical slices from *Fmr1* KO mice following optogenetic activation of local circuits. These studies were consistent with *in vivo* studies of resting and sound driven activity and showed increased synchrony between layers 2/3 and 5. Considering that subcortical inputs are absent in slice electrophysiological studies, these data indicate local cortical deficits or reflect compensatory plasticity of intrinsic properties during the development of the mice from which slices were taken. To investigate the contribution of local cortical deficits *in vivo*, Lovelace et al. (47) examined the effects of *Fmr1* deletion only from excitatory neurons in the forebrain using the *Nex1* promoter. In this mouse model of FXS, FMRP expression was normal in the midbrain and thalamus, while cortical excitatory neurons showed loss of FMRP allowing for an examination of local cortical abnormalities following FMRP loss. EEG resting gamma power, and non-phase locked power in sound-evoked trials were elevated, as seen in global *Fmr1* KO mice. However, the chirp-induced gamma synchronization (ITPC) was normal. These data indicate that a mixture of local cortical processing deficits and inherited deficits from subcortical sites lead to the observed cortical phenotypes, pointing to the need for a balanced investigation across the auditory neuraxis. Indeed, very little is known about subcortical auditory responses in humans with FXS. Interestingly, hyperactive locomotor behavior, but no changes in anxiety-like behaviors, was observed in mice with forebrain excitatory-specific *Fmr1* deletion, pointing to combined cortical and subcortical contributions to behavioral deficits in FXS.

### Cellular Mechanisms of Auditory Hypersensitivity in *Fmr1* KO Mice—The MMP-9 Link

Delving more into the cellular mechanisms of abnormal cortical responses, several studies reported abnormal development and function of specific GABAergic neuron subtype parvalbumin (PV)-expressing interneurons. In particular, PV inhibitory interneurons in the cortex have been implicated in sensory hypersensitivity and abnormal sensory processing in *Fmr1* KO mice in both visual and somatosensory systems (77–79). Gibson et al. (61) found a significant reduction in local excitatory drive on fast-spiking interneurons (putative PV neurons) in layer 4 of the somatosensory cortex. PV-expressing interneurons provide synchronous inhibition of multiple neighboring pyramidal cells, a process that is thought to be important in the generation of the narrowband gamma frequency rhythm (80–82). These cells may also be involved in desynchronizing higher frequency broadband gamma activity, implicating PV cells in the observed EEG phenotypes in FXS (83, 84). A characteristic structural feature of PV cells in the cortex is the preponderance of a specialized extracellular matrix structures called the perineuronal nets (PNNs) (59). PNNs are thought to increase excitability of PV cells (85) and thereby increase network inhibition. PNNs formation around PV cells also coincides with the closure of critical period plasticity windows in sensory cortices (86–89).

Auditory cortical hyperexcitability in FXS may arise from abnormal development of PV cells and PNNs during the P14–P21 window, the time window of divergence in cortical responses in *Fmr1* KO mice (59). A reduced density of PV-expressing cells and the numbers of PNN-enwrapped PV cells in the *Fmr1* KO mouse cortex at P21 may affect PV cell function and cortical inhibition. PNNs are dynamic structures and can be degraded by the activity of multiple proteases, including matrix metalloproteinase-9 (MMP-9). MMP-9 is a zinc-dependent endopeptidase that is found in many cell types, including neurons and glia (90). Among a large family of MMPs, MMP-9, MMP-2, and MMP-3 are widely expressed in the CNS and the expression of MMP-9 is regulated during development (90).

MMP-9 is a translational target of FMRP (91) and in the absence of FMRP, there is increased activity of MMP-9 across multiple brain regions and developmental periods in *Fmr1* KO mice (59, 92, 93). Increased MMP-9 levels and activity were also observed in FXS human samples (92, 94). In addition, neural circuit deficits in *Drosophila* model of FXS were linked to MMPs and removal of *mmp1*, that encodes a secreted form of mmp in drosophila, ameliorated synaptic architecture defects at the neuromuscular junctions of *dfmr1 null* mutants (95). While reduction or loss of MMP-9 expression in *Fmr1* KO mice reduced FXS-like symptoms (59, 92), MMP-9 overexpression in mice resulted in FXS-like symptoms (94). To test the role of MMP-9 in abnormal PV and PNN development, Wen et al. (59) utilized a genetic approach allowing to reduce MMP-9 to the normal levels in the *Fmr1* KO mice. In these mice, not only PNNs were restored to normal levels, in particular around PV-expressing cells, cortical tone-driven responses were also normalized. In addition, abnormal sensory gating as tested with the pre-pulse inhibition of acoustic startle was also improved in these mice (93). Interestingly, even a complete removal of MMP-9 in the *Fmr1* KO mice improved ERP habituation (56). The effectiveness of minocycline treatment in normalizing abnormal ERP habituation in FXS humans was also linked to the reduction of MMP-9 activity (17, 96), suggesting that elevated levels of MMP-9 may contribute to auditory hyperexcitability in FXS. Increased cortical MMP-9 activity and abnormal PV/PNN development were also observed in forebrain excitatory neuron-specific *Fmr1* KO mice (47), suggesting a key role of cortical excitatory neurons in the dysfunction of PV interneurons, enhanced MMP-9 activity and abnormal PNN development. The loss of FMRP in excitatory neurons lead to reduced excitatory innervation of PV cells (61) and PNN loss *via* enhanced MMP-9 activity (47), both of which can affect PV cell functions and cortical inhibition resulting in EEG gamma band abnormalities. Consistent with the role of PV hypofunction in cortical hyperexcitability, enhancing PV cell function in the visual cortex of *Fmr1* KO mice corrected orientation tuning of excitatory neurons and improved mouse performance in a visual perceptual learning task (78).

### Therapeutics to Reduce Sensory Hypersensitivity

Given the strong evidence linking dysregulation of MMP-9 activity to the development of auditory cortex hyperexcitability, this pathway may serve as a potential therapeutic target to reduce sensory hypersensitivity. Minocycline is an FDA-approved antibiotic and a known inhibitor of MMP-9. Minocycline treatment in humans with FXS improved ERP habituation responses (17), and open label studies have shown significant functional improvements in FXS (97). A randomized placebo-controlled study of minocycline showed improvement in Clinical Global Impression Scale compared to placebo and greater improvement in anxiety and mood-related behaviors on the Visual Analog Scale (98).

Several studies have also shown benefits of minocycline treatment in the mouse and the drosophila models of FXS (95). For example, both minocycline treatment and genetic reduction of MMP-9 normalized the rate of ultrasonic vocalizations in *Fmr1* KO mice when paired with a receptive female (99, 100). Minocycline reduced audiogenic seizures, hyperactivity and anxiety-like behaviors in both young and adult *Fmr1* KO mice, but the effects lasted longer when the treatment was given at a young age (101). In contrast, adult mice had to be treated continuously for sustained benefits. These data point to an important element of treatment design –age of administration.

In addition to the improvements in mouse behaviors, a 10-day treatment of adult *Fmr1* KO mice with minocycline also influenced EEG phenotypes (47). By testing resting EEG, ERPs, auditory steady state and chirp response ITPC and non-phase locked power, this study found beneficial effects of minocycline over vehicle treatment in all phenotypes, except resting gamma EEG power. Minocycline treatment increased gamma synchronization in response to auditory stimuli, and reduced sound-evoked power of auditory ERPs in *Fmr1* KO mice compared to vehicle treatment. Although resting gamma power was reduced by minocycline, it was also reduced by vehicle treatment. Because minocycline has multiple targets besides MMP-9, including apoptotic pathway and microglia, it is necessary to test more specific inhibitors. Toward that goal, Pirbhoy et al. (52) tested acute treatment with SB-3CT, a MMP2/9 inhibitor, and demonstrated improved ITPC to auditory stimuli, enhanced PNN formation, and increased PV levels and TrkB phosphorylation in the auditory cortex of *Fmr1* KO mice. Importantly, the reduction of MMP2/9 activity also improved mouse behavior as tested in the open field and elevated plus maze. Good sensitivity and reproducibility of EEG recordings provide a scientific justification for future use of EEG outcome measures in pre-clinical studies, including translationally relevant MEA EEG recordings. Jonak et al. (54) showed that an orally active phosphodiesterase 10A (PDE10A) inhibitor (14-day treatment) normalized the chirp ITPC in *Fmr1* KO mice even at a low dose (0.5 mg/kg) without causing any sedation or effects on baseline EEG power. Taken together, these data indicate that sound-evoked EEG responses may be more sensitive measures, compared to resting EEG measures, to isolate drug effects from placebo in humans with FXS. Minocycline or other MMP-9 inhibitors show much promise in reducing sensory issues in FXS and selecting sensitive outcome measures based on the mouse EEG data may prove useful in designing statistically powerful clinical trials.

Kulinich et al. (55) also explored a non-pharmacological approach in reducing sensory hypersensitivity, in particular, therapeutic effects of reduced sound exposure during the P14–P21 developmental period, when auditory cortical hyperexcitability was first observed. Surprisingly, development of *Fmr1* KO mice in a sound-attenuated environment did not reduce abnormal phenotypes, and in some cases exacerbated the symptoms (55). However, cortical correlates of auditory hypersensitivity were reduced when the mice were exposed to repeated tones at a rate of 5 Hz during this developmental window. Development of PV cells and PNNs, dendritic spines, TrkB phosphorylation and ERP amplitudes were normalized following the developmental sound exposure. These data suggest that developmental sound exposure during the critical period window, and not sound attenuation, may serve as a potential treatment option either alone, or in combination with pharmacological approaches.

### Summary—Quadruple Hit Model of Auditory Hypersensitivity in FXS

Auditory hypersensitivity is a highly debilitating and commonly associated condition in humans with FXS (102). The *Fmr1* KO mouse model of FXS also shows this behavioral phenotype providing a strong basis for examining mechanisms that may help to develop new therapeutic approaches in humans. At a functional level, the remarkable similarities in EEG phenotypes are evident across humans and rodents, including increased gamma band resting power, reduced phase locking to time varying and steady state auditory stimuli but increased non-phase locked power, increased ERP amplitude and reduced habituation of ERPs to repeated stimuli (Table 1). The specificity, reproducibility and sensitivity of these EEG measures provide a strong rationale for using EEG outcomes in pre-clinical trials in mice. Importantly, the scalability and clinical correlations in human EEG work supports widespread use of similar EEG outcomes in clinical studies to see real-world benefits in humans with FXS.

Based on studies of the circuit mechanisms underlying auditory hypersensitivity in FXS, we emphasize a “quadruple hit” model to explain auditory hypersensitivity: (1) individual cortical neurons are hyper-responsive to sounds (59, 60); (2) more cortical neurons respond synchronously to the same sound (60); (3) habituation of cortical neurons to repeated/continuous sounds is reduced (56); (4) background cortical activity is increased (46, 49, 50). These four phenotypes create a milieu of background noise, particularly manifesting as elevated broadband gamma noise, above which cortical neurons need to increase their responses to improve signal to noise ratio in information transfer. From a cellular and molecular perspective, recent studies from our and other groups implicated MMP-9 and PV-expressing inhibitory interneurons in abnormal circuit functions that underlie cortical hyperexcitability (47, 52, 59) as follows:

Loss of FMRP → Increased MMP-9 → Reduced PNNs around PV cells → Reduced excitability of PV cells → Reduced inhibition of cortical networks → Abnormal gamma synchrony and cortical hyperexcitability.

### Future Studies

1. The functional deficits in sensory processing may emerge during specific developmental windows due to abnormal changes in circuit development providing an opportunity to target specific circuits for treatments during these windows (36). Given the vast literature on the critical role of developmental sensory experience that shapes brain structure and function over the lifespan, it is highly likely that early developmental treatments to normalize sensory circuit development will be most effective. However, it remains to be tested whether early developmental therapeutic interventions can normalize sensory processing with long-lasting benefits. It is also unclear whether early postnatal interventions to normalize sensory processing will have broader impacts and prevent abnormal behaviors in humans with FXS, such as anxiety, impaired social communication, delayed language function, and hyperactivity. Early reversal of sensory processing deficits may result in broad-acting benefits, an idea that remains untested in FXS.
2. There is a significant number of molecular targets considered as a treatment for FXS (103, 104). However, clinical trials have either failed or are inconclusive (105), contributing to mounting frustration in the FXS community. While very recent studies using phosphodiesterase inhibitors are promising (54, 106), continued efforts to understand how multiple pathways implicated in FXS interact, leading to circuit dysfunction and abnormal behaviors. One earlier theory suggests enhanced mGluR5-dependent protein synthesis in the *Fmr1* KO mouse model providing a possible link between over-activated mGluR5 and enhanced protein translation in neurons lacking FMRP (107). A recent study also showed a new link between mGluR5 and MMP-9 reporting that deleting or blocking mGluR5 can decrease MMP-9 activity resulting in an elevated (almost doubling) number of PNNs in the somatosensory cortex (108). These data suggest that the increase in mGluR5 activity can lead to increased MMP-9 activity and PNN loss in FXS, suggesting a potential link between the mGluR5 and MMP-9 theories of FXS hyperexcitability. Future studies should explore these links in greater detail to determine whether MMP-9 acts downstream of mGluR5 and can be targeted therapeutically alongside or instead of mGluR5 antagonists, helping to reduce any buildup of tolerance and side-effects.
3. There is a predominant focus on the neocortex and hippocampus in studies of FXS and ASD, which is particularly true in humans. However, our investigations of the mechanisms of auditory dysfunction in FXS indicate that the cortically recorded phenotypes may reflect a mixture of local circuit deficits and subcortical deficits. A systematic investigation of deficits in subcortical processing and their developmental time course using transgenic mouse lines and promoters that allow spatial and cell-type-specific deletion or re-expression of FMRP could facilitate these studies in animal models. In humans, frequency following responses (FFR) which likely originate in the midbrain/brainstem region (109, 110) can be recorded to identify differential subcortical processing in FXS and ASD.
4. The ability of early sound exposure, but not sound attenuation, to reduce cortical hyperexcitability symptoms suggests that developmental trajectories of atypical sensory processing need to be investigated across closely spaced developmental ages. Examination of deficits at a single age or a small number of ages may miss the main cause of pathology early on, and only record manifestation of compensatory mechanisms (32, 33), which may be indirectly altered by the genetic mutation and can be beneficial (111–113).
5. The excitement around developments in the field of gene therapy indicates this approach may allow re-expression of FMRP in the near future (114, 115). However, our understanding of the function of FMRP at different ages remains underwhelming. In particular, it is unclear whether adults may benefit from FMRP re-expression, or if re-expression has to occur during embryonic or early postnatal development. There is no study comparing the developmental vs. adult effects of FMRP expression in the same model, using the same outcome measures. One published paper on this topic showed that acute expression of FMRP in adult prefrontal cortex is sufficient to elicit normal learning of adult *Fmr1* KO mice in a prefrontal cortex dependent task (116). Despite the strong evidence for early developmental abnormalities in FXS, whether targeted interventions at this age provide long-lasting benefits is also unclear. In Angelman Syndrome, reactivation of Ube3A at different developmental time points has a phenotype-specific effect, but in Rett Syndrome benefits are seen for both early and late corrections of the deficits (117–119). These data from other forms of ASD indicate that a systematic study of effects of FMRP re-expression at different ages, and using a broad range of structural, functional and behavioral outcome measures is necessary. The findings reviewed here indicate that studies of sensory hypersensitivity may provide a tangible and translationally relevant niche to address these urgent issues.

## Author Contributions

KR, DB, and IE contributed to the writing of the review. All authors contributed to the article and approved the submitted version.

## Funding

This work was supported by the National Institute of Child Health and Human Development and the National Institute of Mental Health, U.S. Army Medical Research and Materiel Command and FRAXA Research Foundation.

## Conflict of Interest

The authors declare that the research was conducted in the absence of any commercial or financial relationships that could be construed as a potential conflict of interest.

## Publisher's Note

All claims expressed in this article are solely those of the authors and do not necessarily represent those of their affiliated organizations, or those of the publisher, the editors and the reviewers. Any product that may be evaluated in this article, or claim that may be made by its manufacturer, is not guaranteed or endorsed by the publisher.

## Abbreviations

ASD: Autism Spectrum Disorders
Fmr1: Fragile X Mental Retardation 1 gene
FMRP: Fragile X Mental Retardation Protein
FXS: Fragile X Syndrome
EEG: Electroencephalography
ERP: Event Related Potential
ITPC: Inter-Trial Phase Coherence
KO: Knock Out
MMP-9: Matrix Metalloproteinase-9
P: Post-natal day
PNN: Perineuronal Nets
PV: Parvalbumin
SPD: Sensory Processing Disorder
STG: Superior Temporal Gyrus
STP: Single Trial Power
WT: Wild Type.
